# Supplementary material for: The effects of a yoga intervention on balance and flexibility in female college students during COVID-19: A randomized controlled trial
Source: PLoS One. 2023 Mar 22;18(3):e0282260. doi: 10.1371/journal.pone.0282260 (PMC10032532; doi:10.1371/journal.pone.0282260)
Supplement: S1 File — (DOC) [file pone.0282260.s001.doc]

**Recruiting participants for the yoga intervention health study**

**[Experimental content]**

Effects of yoga intervention on balance and flexibility of female college students during COVID-19.

**[Experiment in brief]**

The COVID-19 pandemic poses a serious health threat to female college students and deserves more attention. This study is a randomized controlled trial conducted during the COVID-19 pandemic. The purpose of this trial was to explore whether yoga would positively affect their balance and flexibility and whether yoga could be used as a primary form of home exercise for female college students to help them stay healthy during the COVID-19 outbreak.

**[Location of experiment]**

Yoga classroom of Chengdu Normal University.

**[Participant Requirements]**

1. Female university students in school.

2. Have not practiced yoga before.

3. Do not perform other exercises during the experiment.

4. Serious attitude toward attending yoga class on time.

5. Voluntarily participate in this study and sign the informed consent form.

**[Exclusion criteria]**

1. Having a chronic illness or physical disability.

2. Current or previous yoga experience.

3. Physical condition unsuitable for yoga.

4. Physical conditions unsuitable for sports training.

5. Hypertension, hypoglycemia, cerebellar disease, joint disease or eye disease.

**[Experiment time]**

From April 1, 2020, to July 31, 2020, every Wednesday and every Saturday evening from 18:00-19:10.

**[Participants' Compensation]**

Participants who participate in the yoga practice on time and persist in completing all the experimental tasks will be paid 200 RMB each class, which will be distributed through Alipay after the study is completed.

**-- Enrollment Method --**

If you meet the above criteria and are willing to participate in this research project, please contact 13438917548 (same mobile and WeChat number).
