# Supplementary material for: The effects of a yoga intervention on balance and flexibility in female college students during COVID-19: A randomized controlled trial
Source: PLoS One. 2023 Mar 22;18(3):e0282260. doi: 10.1371/journal.pone.0282260 (PMC10032532; doi:10.1371/journal.pone.0282260)
Supplement: Attachment — Submitted filename: Response to Reviewer Comments.doc [file pone.0282260.s003.doc]

Dear Xin Ye, Ph.D. Academic Editor, Reviewer 1 and 2:

Thank you for your comments and suggestions on the manuscript. The comments and suggestions are all valuable and very helpful for revising and improving our study, as well as the important guiding significance to our research. We have studied the comments carefully and have corrected this revised manuscript. Thank you very much! Thank you very much for giving us this valuable opportunity to revise! We now respond to each of your questions, and resubmit the revised manuscript.

**1. Reviewer #1:** This randomized controlled study examined the effect of yoga exercise on balance and flexibility in female college students. The author found that yoga would be a good alternative exercise for those who want to maintain exercise while improving balance and flexibility. Generally, yoga is good for improving flexibility and balance, which has been shown in many previous studies. The author focused on female college aged students because they deal with a variety of challenges such as academic and employment challenges that can have an impact on their physical balance and flexibility. The manuscript needs better organization and clarity. It seems the author has missed an opportunity to explore a variety of important factors affecting flexibility and balance in this study and enhance the data beyond what is known. Please underscore the scientific value-added to your paper in your abstract. Your abstract should clearly state the essence of the problem you are addressing, what you did and what you found and recommend. That would help a prospective reader of the abstract to decide if they wish to read the entire article.

**1. Answer:** Thanks for your valuable suggestions. We have revised the content of the manuscript as a whole and reformulated the title, “The effects of a yoga intervention on balance and flexibility in female college students during COVID-19: A randomized controlled trial”.

The innovations of this paper are as follows: First, this study is a randomized controlled experiment carried out during the COVID-19 pandemic. Female college students are seriously affected by COVID-19. It is of great significance to study the impact of a yoga intervention on the balance ability and flexibility of female college students. This study is conducive to providing a reference for the implementation of timely and effective health interventions and promoting health maintenance for female college students during the COVID-19 pandemic. Second, most studies use the sit and reach test index to measure participant flexibility. In addition to the sit and reach test index, this study also adds the splits test index to measure the flexibility of the participants' groins and hips, expanding the selection range of flexibility measurements to more comprehensively assess the flexibility of the participants.

The significance and value of this study is that during the outbreak of COVID-19, female college students are more likely to have anxiety, tension and other stress reactions to the COVID-19 epidemic, and these negative emotions have a serious impact on their health. We should focus on the physical and mental health of female college students and help them overcome difficulties through timely and effective sports intervention. For example, starting with the conclusions of this study, we can take yoga as the main method of home exercise, design special online yoga courses and activities, and give greater care and support to female college students to encourage their active participation in yoga, alleviate their negative emotions during the outbreak of COVID-19, and improve their health.

The abstract has also been revised accordingly as you suggested.

We have polished the language of the manuscript, so please read the revised version of this submission. Thank you so much!

**2. Reviewer #2:** The randomized controlled study looking at the effects of 16-week yoga intervention on balance and flexibility in college women. To my knowledge, numerous research has shown that yoga is effective in improving balance and flexibility in young women. The study measurements are simple. Additionally, the statistical analysis is flaw. For example, the author used paired sample t-test and independent sample t-test multiple times to compare the effects of yoga in flexibility and balance in the 2 groups. However, multiple t-tests on the same data will result in addictive Type I errors. The correct method is to use the two-way mixed ANOVA. In summary, I cannot recommend accepting this manuscript.

**2. Answer:** These are good questions. Thank you so much! We have revised the manuscript based on the issues you raised, so please read the latest revision of this submission.

The innovations of this paper are as follows: First, this study is a randomized controlled experiment carried out during the COVID-19 pandemic. Female college students are seriously affected by COVID-19. It is of great significance to study the impact of a yoga intervention on the balance ability and flexibility of female college students. This study is conducive to providing a reference for the implementation of timely and effective health interventions and promoting health maintenance for female college students during the COVID-19 pandemic. Second, most studies use the sit and reach test index to measure participant flexibility. In addition to the sit and reach test index, this study also adds the splits test index to measure the flexibility of the participants' groins and hips, expanding the selection range of flexibility measurements to more comprehensively assess the flexibility of the participants.

The statistical methods used in the manuscript have also been modified, and repeated measure analysis of variance is used in this study.

In this study, an analysis of variance (ANOVA) with a mixed design of 2 (group: yoga group, control group) × 2 (time: pretest, posttest) was used to analyze the effects of yoga intervention on the balance ability and flexibility of female college students seriously affected by COVID-19 and to verify whether yoga can be used as a primary method of home exercise for female college students while having a positive impact on female college students in the COVID-19 epidemic period.

Thank you for your very helpful comments and advice! If the manuscript still needs to be revised, please do not hesitate to contact us. Thank you very much!

Kind regards,

Xue Luo and Xu Huang
